# The contribution of co-reference resolution to supervised relation detection between bacteria and biotopes entities

**DOI:** 10.1186/1471-2105-16-S10-S6

**Published:** 2015-07-13

**Authors:** Thomas Lavergne, Cyril Grouin, Pierre Zweigenbaum

**Affiliations:** 1 LIMSI-CNRS, 91405 Orsay, France; 2 Université Paris-Sud, 91405 Orsay, France

## Abstract

**Background:**

The acquisition of knowledge about relations between bacteria and their locations (habitats and geographical locations) in short texts about bacteria, as defined in the BioNLP-ST 2013 Bacteria Biotope task, depends on the detection of co-reference links between mentions of entities of each of these three types. To our knowledge, no participant in this task has investigated this aspect of the situation. The present work specifically addresses issues raised by this situation: (i) how to detect these co-reference links and associated co-reference chains; (ii) how to use them to prepare positive and negative examples to train a supervised system for the detection of relations between entity mentions; (iii) what context around which entity mentions contributes to relation detection when co-reference chains are provided.

**Results:**

We present experiments and results obtained both with gold entity mentions (task 2 of BioNLP-ST 2013) and with automatically detected entity mentions (end-to-end system, in task 3 of BioNLP-ST 2013). Our supervised mention detection system uses a linear chain Conditional Random Fields classifier, and our relation detection system relies on a Logistic Regression (aka Maximum Entropy) classifier. They use a set of morphological, morphosyntactic and semantic features. To minimize false inferences, co-reference resolution applies a set of heuristic rules designed to optimize precision. They take into account the types of the detected entity mentions, and take advantage of the didactic nature of the texts of the corpus, where a large proportion of bacteria naming is fairly explicit (although natural referring expressions such as "the bacteria" are common). The resulting system achieved a 0.495 F-measure on the official test set when taking as input the gold entity mentions, and a 0.351 F-measure when taking as input entity mentions predicted by our CRF system, both of which are above the best BioNLP-ST 2013 participant system.

**Conclusions:**

We show that co-reference resolution substantially improves over a baseline system which does not use co-reference information: about 3.5 F-measure points on the test corpus for the end-to-end system (5.5 points on the development corpus) and 7 F-measure points on both development and test corpora when gold mentions are used. While this outperforms the best published system on the BioNLP-ST 2013 Bacteria Biotope dataset, we consider that it provides mostly a stronger baseline from which more work can be started. We also emphasize the importance and difficulty of designing a comprehensive gold standard co-reference annotation, which we explain is a key point to further progress on the task.

## Background

Scientific documents provide useful information in many domains: an example is microorganism ecology, which involves a variety of microorganisms (bacteria, living and dead cells, etc.) and habitats (food, medical, soil, water, hosts, etc.) that have been described in detail in the literature. However, reading the huge amount of articles published nowadays is too time-consuming for a human. Natural Language Processing (NLP) techniques have therefore been designed to process these documents quickly and make the extracted information available for further studies.

The identification of mentions of bacteria and biotopes in scientific texts has been addressed during the last two BioNLP *Bacteria Biotopes *shared tasks [1-4]. Detecting mentions of microorganisms and habitats is indeed a first step to access the semantic content of these texts. Identifying relationships between the detected entities is the next step to represent the knowledge conveyed by a text more completely. This is the topic of the present work.

The model emphasized here, as proposed by the Bacteria Biotopes task organizers, is that of knowledge acquisition from texts. This differs from information extraction in the following respect: instead of focusing on each mention of an entity in a text and each occurrence of a relation between two entity mentions, it takes a step back to look at entities and their relations. This has two important consequences:

• As emphasized by the organizers [2-4], this requires to detect which mentions refer to the same entity, i.e. entertain a *co-reference relation*. This can be modeled as co-reference chains, i.e. equivalence classes of mentions which all co-refer to the same entity.

• Relations must be found at the level of entities, i.e. at the global level of full co-reference chains instead of individual entity mentions.

Co-reference has been addressed in information extraction as far back as in the MUC-6 evaluation campaign [5]. An anaphora is a dependence relation between two referential expressions, while co-reference holds among referential phrases that refer to the same referent in a text; a co-reference chain collects the full set of co-referential phrases and can include anaphora and/or co-references (adapted from [6]).

Co-reference resolution consists in finding in a text all expressions referring to a given entity, regardless of the surface forms of these expressions [7]. It has been identified as an important need for information extraction both in clinical texts [8-11] and in the scientific literature, including molecular biology [12-15]. Protein co-reference resolution was proposed as a supporting task (COREF) in the BioNLP Shared Task 2011 [16]. In this purpose, co-reference relations were annotated in a training corpus and provided to the participants. The best participant system [17] reused and extensively adapted the existing co-reference resolution framework Reconcile [18].

Several other systems [19-21] used syntactic parsers and rules to detect co-reference relations. They follow a similar overall schema: determine the set of anaphors (mostly pronouns and definite noun phrases) and potential antecedents (most noun phrases) based on the results of syntactic analysis; then given an anaphor, the rules enforce the compatibility of antecedent candidates with the anaphor and manage a notion of salience or discourse preference. The selection of the best antecedent candidate is done in a sieve-like manner (in [20,21], as in [22]) or through a score [19].

However, after the 2011 Shared Task, [21] designed a rule-based method based on domain-specific information, whose minimal configuration outperformed the participant systems. They emphasize the difficulty to transfer such knowledge across domains. The organisers of the Bacteria Biotope task stressed in both the 2011 and 2013 BioNLP Shared Tasks that co-reference resolution was important to detect Bacteria and Biotope relations [2,3,23]. We too hypothesize that co-reference resolution should be important to acquire knowledge about relations between bacteria and their locations in the BioNLP-ST 2013 Bacteria Biotope (BB) task. Despite this general opinion, according to the BioNLP-ST 2013 workshop proceedings, no participant in the 2013 BB task investigated how co-reference impacts this task. This is the focus of this paper.

Our general strategy has been to start from state-of-the-art methods, combine them and optimize them to address the Bacteria Biotope requirements, to obtain an evaluation of their worth in the framework of the present task. Therefore, whereas the choice of features that we made is specific to the task, the strategy used to determine these features, create the system, optimize it and evaluate it should be general and applicable to a wide range of tasks.

This paper presents the following contributions:

• An end-to-end system for mention and relation detection in the Bacteria Biotope corpus;

• The design of a co-reference resolution component meant to help relation detection in this task, and its evaluation against the gold co-reference annotations provided with the corpus;

• A study of the impact of co-reference resolution on relation detection.

The system has state-of-the-art performance compared to BioNLP-ST 2013 participant systems, which it outperforms when co-reference resolution is applied.

**Figure 1:**
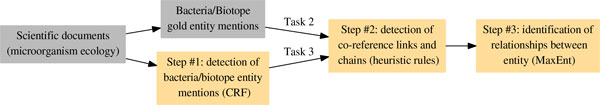
**Global view of the process**.

Figure 1 gives a global view of the process we designed to handle the identification of relationships between entity mentions of bacteria and their biotopes.

In the remainder of this paper, we introduce the corpus provided for the BioNLP-ST 2013 Bacteria Biotope task. We then present the methods we designed to perform mention identification, co-reference resolution, and relationship identification. We report and discuss the results obtained on each of these three sub-tasks after the official challenge, and conclude with our views on requirements for further progress on this task.

## Corpus presentation

The corpus comprises web pages about bacterial species written for non-experts. Each text consists of a description of individual bacterium and groups of bacteria, in terms of first observation, characteristics, evolution and biotopes. The corpus is split into three sub-corpora: training corpus (52 documents), development corpus (26 documents), and test corpus (53 documents, among which 27 were used for the entity mention identification task and 26 for the relation identification task).

Table 1 describes global statistics on the training and development corpora and the distribution of annotations over categories. Three types of entities are annotated; *Habitat *and *Bacteria *categories are the most represented (each type encompasses 45 to 50% of all annotations). While the *Geographical *type has a small number of annotations, a corpus study revealed that instances of this type exhibit different subtypes: countries, facilities, organizations, etc., making it difficult to detect all mentions belonging to this type.

**Table 1:** Gold annotation statistics on training and development corpora

		Training	Development
Global statistics	# Documents	52	26
	# Words	16,294	9,534
	
	Avg # words/document	313.3	366.7

Entity mentions	# Bacteria	832 (44.8%)	515 (42.8%)
	# Habitat	934 (50.3%)	611 (50.8%)
	# Geographical	91 (4.9%)	77 (6.4%)

Relations	# Localization	596 (79.5%)	434 (84.3%)
	# PartOf	154 (20.5%)	81 (15.7%)
	
	# Equiv classes	133	73

Gold annotation statistics on training and development corpora.

Two types of relationships between entities occur in this corpus: a *Localization *relation (the main relation in this corpus, about 80% of all annotated relationships) and a *PartOf *relation. The localization relation occurs between a bacterium (*Bacteria *type) and a host (either *Habitat *or *Geographical *type) while the PartOf relation occurs between two entities of the same type.

**Figure 2:**
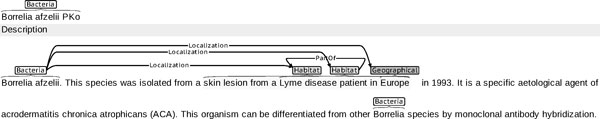
**Annotated corpus excerpt**. Excerpt from the annotated corpus (BTID-10087 file, training corpus) using the BRAT Rapid Annotation Tool.

Figure 2 shows an excerpt of the annotated corpus (gold standard training corpus). This excerpt displays:

• Six annotated entity mentions belonging to the three entity types:

**- ***Bacteria*: "Borrelia afzelli PKo", "Borrelia afzelli" and "Borrelia";

**- ***Habitat *: "skin lesion from a Lyme disease patient in Europe" and "Lyme disease patient in Europe";

**- ***Geographical *: "Europe".

• Three *Localization *relationships between:

**- ***Bacteria *and *Habitat *mentions: "Borrelia afzelli" (bacterium) located in "skin lesion from a Lyme disease patient in Europe" and "Lyme disease patient in Europe" (localizations);

**- ***Bacteria *and *Geographical *mentions: "Borrelia afzelli" (bacterium) and "Europe" (localization).

• A *PartOf *relation between two *Habitat *mentions: "Lyme disease patient in Europe" (host) and "skin lesion from a Lyme disease patient in Europe" (part).

**Figure 3:**
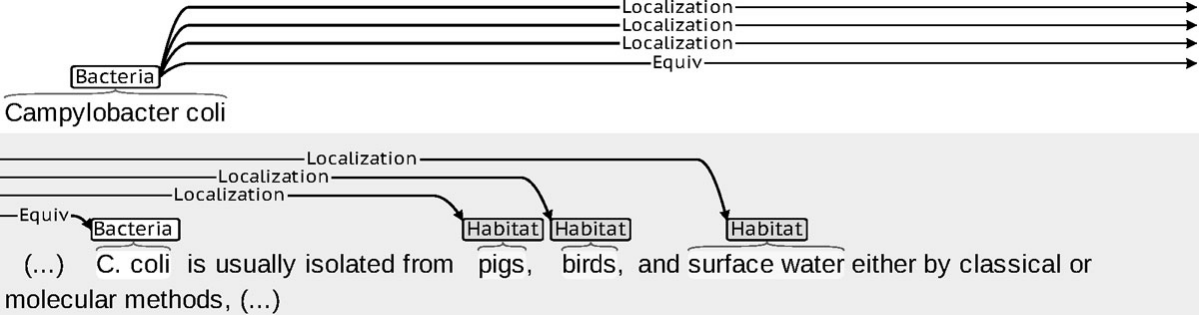
**Co-reference between similar Bacteria mentions**. Co-reference relation between graphically similar Bacteria entity mentions ***Campylobacter coli ***and ***C. coli***: these mentions and this instance of co-reference relation were given in the gold standard annotations provided with the training corpus of Task 2. This co-reference relation is instrumental in the detection of the Localization relations between ***Campylobacter coli ***and its habitats ***pigs***, ***birds***, and ***surface water***. Ellipsis ***(...) ***shows skipped material.

Figure 3 shows another excerpt from the same corpus, in which a co-reference relation (*Equiv*) is annotated. Bacteria entity mention *Campylobacter coli *is later abbreviated as *C. coli*. Both are annotated as Bacteria mentions, and the fact that they both refer to the same entity is marked by the co-reference link *Equiv*. Since only *C. coli *is explicitly said to be located in a series of habitats (*pigs, birds*, and *surface water*), the co-reference relation is instrumental in the detection of the gold standard Localization relations between these habitats and *Campylobacter coli*: relation detection can operate more easily within the sentence where both *C. coli *and its habitats, e.g., *pigs*, occur. Then, following the co-reference link, the Localisation relation *Campylobacter coli −*(loc)*→pigs *can be returned using the most explicit designation for the bacterium, which is preferred according to the task guidelines.

Moreover, as explained in the Methods section (subsection Relationships identification), the gold standard co-reference relations are used by the official relation scoring system to conflate co-referring mentions when comparing a gold relation annotation and a system relation annotation. Knowing which mentions are thus considered as equivalent is therefore all the more important.

**Figure 4:**
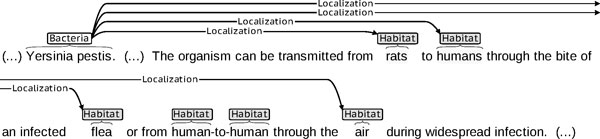
**Co-reference through definite noun phrase anaphora to a Bacteria-type mention**. Co-reference relation through definite noun phrase anaphora to a Bacteria-type mention: ***The organism ***refers to Bacteria-type mention ***Yersinia pestis***. Since ***The organism ***is not the name of a bacterium, it was not annotated in the gold standard annotations provided with the training corpus of Task 2, nor was the corresponding co-reference relation. Since sentence ***The organism ... infection ***asserts the locations where ***The organism ***can be found, it is important to capture the co-reference between ***This organism ***and ***Yersinia pestis ***to detect the Localization relations between this bacterium and its habitats. Ellipsis ***(...) ***shows skipped material.

Figure 4 shows yet another example found in the training corpus. *The organism *refers to the Bacteria mention *Yersinia pestis*. The sentence where *The organism *occurs expresses that it is found in specific habitats (*rats, humans*, etc.). In contrast to the example in Figure 3, *The organism *is not the name of a bacterium, and per the task annotation guidelines is not considered as a Bacteria entity mention. It is hence not included in the gold mention annotations, which entails that it cannot be involved in a co-reference relation annotation. Independently of the task annotation guidelines, *The organism *does indeed refer to *Yersinia pestis*: recovering this mention and its co-reference link to *Yersinia pestis *will provide the missing link to detect its Localization relations in this sentence.

## Methods

In general, given a specific task, two broad strategies can be distinguished: (*i*) use a very generic framework or system and apply it to the specific task at hand; this is the choice implemented for instance by [24], who applied the same system, with minimal specific extensions, to all the BioNLP 2013 tasks; (*ii*) extend a combination of state-of-the-art methods by finely tuning them to this task; we opted for this second strategy, assuming that it has better potential to fit the task and obtain better results.

We process each document in three steps implemented in three modules (see Figure 1). In the end-to-end system (Task 3), each module takes as input the results of the preceding modules. When gold mentions are provided (Task 2), the pipeline starts with Step 2.

1. Mention identification takes as input a text and produces entity mentions, each with its span and type;

2. Co-reference resolution produces co-reference chains between these mentions, possibly adding anaphoric expressions that point to some of these mentions; these anaphoric expressions constitute new (indirect) entity mentions;

3. Relation detection produces binary relations between entities; each of the two entities linked by a relation is represented by one of its mentions found at step 1.

To identify entity mentions and to detect relationships between these mentions, we relied on machine-learning methods.

Entity detection is well modelled as a sequence classification task. Conditional Random Fields (CRFs) and Structured Support Vector Machines (SSVMs) are state-of-the-art methods for sequence classification and generally obtain similar results (see e.g. [25]). Since one of the authors [TL] is the author of a very competitive log-linear toolkit which includes CRFs [26], we chose this classifier over others.

Relation detection is modelled as a standard classification task. Among state-of-the-art classifiers, Maximum Entropy (MaxEnt) and Support Vector Machines (SVM) are popular classifiers which obtained good results on previous tasks (e.g., [27,28]). Our log-linear toolkit also covers MaxEnt classification, hence our choice of this classifier for relation detection.

To summarize, we used two distinct formalisms implemented in the Wapiti system [26] to build our models:

• Conditional Random Fields *(CRF) *[29,30] to identify bacteria and biotope mentions;

• Maximum Entropy *(MaxEnt) *[31,32] to detect the relationships between entities.

When introducing the corpus, we explained that co-referring expressions and co-reference relations are not fully annotated in the training and development corpora. This does not provide good conditions to train supervised machine learning methods, and prevented us from trying to adapt an existing trainable system such as Reconcile [17]. Instead, we adopted linguistically-inspired rule-based methods which take advantage of the particular discourse structure and of the particular types of referring expressions of the input texts.

### Mention identification

To identify bacteria and biotope mentions in the text, we used a CRF-based framework we specifically designed for this task [33]. We did not perform any cross-validation but automatic feature selection was carried out through the *l*1 regularization. We built our model using both "classical" internal features (typographic form, presence of digits in the token, presence of a punctuation mark in the token, token length) and a few lexical features:

• Presence of the token in the OntoBiotope Ontology terms http://bibliome.jouy.inra.fr/MEM-OntoBiotope/OntoBiotope_BioNLP-ST13.obo, this resource was provided by the organizers for the purpose of the normalization stage in the first sub-task). This ontology comprises 1,756 concepts from the biotopes domain. In this ontology, each concept has been given a unique ID and is associated with preferred terms and associated synonyms. We noticed that 78.0% of the tokens from both training and development corpora found in the ontology correspond to a habitat name in the reference.

• Presence of the token in the NCBI taxonomy. The NCBI taxonomy (http://www.ncbi.nlm.nih.gov/taxonomy/) [34] describes a small part *(about 10%) *of the living species on Earth, based on public sequence databases. We extracted from this taxonomy all names belonging to the *Bacteria *category (24.3% of the content), resulting in a list of 357,387 bacteria taxa, including a few variants of bacteria names. We noticed that 95.1% of the tokens from both training and development corpora found in this subset of the NCBI taxonomy correspond to a Bacteria name in the reference annotations.

• We used the Cocoa (Compact cover annotator for biological noun phrases http://npjoint.com/annotate.php, annotations were provided by the organizers on the three sub-corpora as part of the supporting resources of the BioNLP 2013 Bacteria Biotopes shared task) annotation categories. Indeed, we observed that a few annotation categories are often tied with one of the three kinds of entities we have to process: *Cell, Chemical, Mutant Organism, Organism, Protein, Unknown *with Bacteria mentions, *Body part, Cell, Cellular component, Chemical, Disease, Food, Geometrical part, Habitat, Location, Multi-tissue structure, Organism, Organism subdivision, Pathological formation, Tissue *with Habitat names and *Company, Habitat, Technique, Unknown *with Geographical names.

• Finally, we also created unsupervised word clusters using Brown's algorithm [35] with Liang's code (https://github.com/percyliang/brown-cluster) [36] on a total amount of 2,015 scientific documents: 131 documents from the training, development and test sub-corpora, and 1,884 new documents from the same sources. These new documents were not used during the challenge. They have been provided by the organizers as part of a distinct research project, for additional experiments based upon the methods we implemented in the BioNLP 2013 shared task. We used the following parameters to build those clusters: creation of a total amount of 120 classes, using all tokens that occur at least two times in the overall corpus, with a maximum depth of three levels during the trees building.

The annotation scheme allows for nested entity mentions, i.e. the span of a mention can be included in the span of another, larger mention. To deal with nested entity mentions, we built a list of the nested mentions found in the training and development corpora. For instance, *respiratory tracts of *animals and *animals *are two Habitat mentions where the latter is nested in the former. We recorded the latter as a mention that can be found within a larger span. We used the recorded list in a post-processing stage: when an occurrence of these bacteria or biotope mentions was found within a mention predicted by the CRF model, we added it as an additional mention.

From the excerpt shown in Figure 2, our pipeline identified the phrases *"Borrelia afzelii PKo" *and *"Borrelia afzelii" *in the NCBI taxonomy; the phrases *"skin lesion", "patient" *and *"Europe" *in the OntoBiotope ontology; and the following phrases were annotated by Cocoa: *"Borrelia afzelii", "species", "patient", "organism" *and *"Borrelia species" *(organism), *"skin lesion" *(pathological formation), *"Lyme disease", "acrodermatitis chronica atrophicans" *and *"ACA" *(disease), *"Europe" *(habitat), *"monoclonal antibody" *(molecule) and *"hybridization" *(process); cluster ID were given to each token using the Brown algorithm. The CRF used those features in combination with surface feature to identify bacteria and biotope entity.

### Co-reference resolution

*Co-reference resolution *over entity mentions consists in identifying on the one hand a *referring expression *(or *anaphor *or *anaphoric expression*), i.e. an expression which co-refers to an already mentioned entity, and its *antecedent*, i.e. a former entity mention with which it co-refers. The term *anaphor *generally specifically applies when the referring expression points at a preceding expression in the text (i.e. when it takes part in an *anaphora*; this is the case of *The organism *in Figure 4), as opposed to a free-standing referring expression such as a proper name which does not need this condition to obtain (this is the case of *C. coli *in Figure 3).

We can summarize as follows the principles driving our approach to co-reference resolution in the present context. Co-reference resolution is addressed here as a means to cluster entity mentions into equivalence classes representing entities, so that relation detection can be managed at the entity level, as detailed in the next section. We consider this requires to emphasize co-reference resolution precision over recall: a false positive co-reference is liable to lead to the incorrect propagation of relations to larger clusters of mentions; whereas a false negative co-reference is likely to split a cluster into two smaller clusters of mentions, possibly propagating a relation to fewer mentions, and only provoking a missed relation if the relation is only expressed once on an anaphoric expression. Therefore our goal here is not to setup the most complete and general co-reference resolution system, but one that will be useful for relation detection in the present corpus, oriented towards a high precision while having a reasonable coverage. Note also that co-reference resolution is only considered here among mentions of the three types of entities addressed in the Bacteria Biotope task, i.e. bacteria, habitats, and geographical locations. This, and other properties described below, makes co-reference resolution in the present corpus and task distinct from previously reported work, e.g., in the BioNLP 2011 COREF task [16].

Some general co-reference resolution systems are available for download. We tried two of the best: Reconcile [18] (http://www.cs.utah.edu/nlp/reconcile/), based on machine learning, has state-of-the-art performance on common co-reference resolution test sets, such as MUC-6, MUC-7, and ACE; and the Stanford Deterministic Coreference Resolution System [37], based on deterministic rules, which was the top ranked system at the CoNLL-2011 shared task. However, because these generic systems do not have specific knowledge about which entity types are targeted in the present corpus, off-the-box application of these systems reveals issues in the determination of entity mentions and attempts to find co-reference relations with other noun phrases in the texts. This makes their results poor for bacteria and biotope co-reference resolution in the present corpus. To illustrate this point, we provide more details on the performance of Reconcile in the Results section. We obtained these results by running Reconcile on the training and development corpora and converting its output to the format expected by the CoNLL-2011 co-reference resolution scorer (details on the scorer are in the Results and Discussion section, subsection Co-reference resolution). As mentioned in the introduction of the Methods section, re-training a system such as Reconcile, as performed in [17], was not an option because of the absence of full co-reference annotations in the training corpus. As a consequence, we designed and implemented a heuristic, rule-based co-reference resolution method which relies on a linguistic observation of the training corpus.

Three types of anaphors can be distinguished in general:

• pronouns (*it, They*);

• definite noun phrases (*the strain*), including those with a possessive adjective (*this bacterium*);

• proper names (*Bifidobacterium, Bradyrhizobium japonicum, B. subtilis*), to which can be added named entities (*Chlorobium phaeobacteroides strain DSMZ 266 T*).

Pronouns are usually the most ambiguous type, and are more likely to incur false positive co-references. For instance, in sentence *[^bacteria^Vibrios] are facultatively anaerobic [^bacteria^bacteria] that are metabolically similar to the [^bacteria^Enterobacteriaceae]. They are ubiquitous to [^habitat^oceans], [^habitat^coastal waters], and [^habitat^estuaries]*., the pronoun *They *could refer either to the first bacterium name *Vibrios*, to the generic name *bacteria*, or to the closest name *Enterobacteriaceae*, making it difficult to choose the correct antecedent. Besides, possessive pronouns (*its, their*) nearly never play a role in the training corpus to help relation extraction. We thus decided not to handle pronouns at all. We return on this choice in the Results section. An important difference between the present task and the COREF task of the BioNLP 2011 Shared Task [16] is that the latter did not address entity mentions as anaphors. In contrast, in the present task, only entity mentions are linked by gold co-reference relations.

Co-reference necessarily obtains between mentions of the same entity type. Because of that, it heavily relies on the former detection and typing of entity mentions in the source text. However, the entity mentions targeted in the entity identification step only cover named entities, including proper names, and some noun phrases (generally bare nouns such as *bacterium*). This means that anaphors of the other types must be detected in the present step. We return to this point later.

We adopted two strategies to handle definite noun phrases and proper names:

**Similarity of form **Co-referring proper names should be identical or differ in a minimal way. An edit distance [38] is a convenient way to compare two strings and ensure that they differ by less than a number *e *of edit operations. Strube *et al*. [39] obtained improvements in the processing of definite noun phrases and proper names by adding a minimum edit distance comparison to their feature set. There are however conventional ways of abbreviating bacterium names, such as writing *B. subtilis *for *Bacillus subtilis*. We therefore implemented the detection of a series of such conventional abbreviations. This strategy is also applicable to some extent to definite noun phrases (e.g. *This strain, The strain*), although in principle the co-reference of such anaphors would better be obtained indirectly through their having the same antecedent bacterium strain (e.g. *Bradyrhizobium japonicum strain USDA110*). Furthermore, we noticed that bacteria mentions containing only the term *bacteria, bacterium *or *bacterial *are very generic; because of that, they seldom carry a relation annotation.

Besides, their co-reference can be fairly ambiguous because such a term can refer to different bacteria in the same text. We therefore decided to block similarity-based co-reference detection for these expressions. Finally, we tested constraints on the maximum hierarchical distance between a mention and its antecedent (e.g., is a genus allowed to co-refer with a strain?): we designed patterns to detect the hierarchical level of each bacterial mention along the cline {strain *<*subspecies *<*species *<*genus *<*family . . . }, and set a maximum hierarchical distance (each jump counting for a distance of one) allowed between two co-referring mentions.

**Actual anaphora resolution **An anaphora occurs when an anaphor points to a preceding expression in the text. Typical examples include the use of pronouns (*They*) and definite noun phrases (*the bacterium*), possibly involving a demonstrative adjective (*this strain*). We provide more detail on our handling of anaphora resolution in the remainder of this section.

We observed in the training corpus that among the three types of entities, anaphora mostly involves bacteria whereas similarity-based co-reference is a prevalent phenomenon among habitat mentions and among geographical locations, and also applies to bacteria. Actually, as presented in the Corpus section above, each document is centered on a bacterium or group of bacteria, in the style of an entry in an encyclopedia. Our anaphora resolution methods therefore exploit the structure this induces on the documents, with one or more bacterium entries and their descriptions. This generates the use of frequent anaphora patterns such as sentence-initial *This bacterium *expressions referring to the current bacterium entry.

Based on a study of the training corpus, we modelled this as follows:

• Centering. We track the *focus bacterium *all along a text. In an approximation of the centering mechanisms [40] at work in text cohesion and anaphora resolution, we consider that the focus bacterium is initially the first bacterium in the document (it is systematically mentioned as the title, i.e. alone on the first line of the input text); in each new paragraph, the first mention of a bacterium becomes the focus bacterium for the paragraph.

• Recentness. We also track the last bacterium mentioned in the text. The combination of Centering and Recentness plays the same role as, e.g., the Salience measure of [19] or the discourse-type rules of [20,21].

• Sentence-initial definite descriptions of bacteria including a demonstrative adjective. As in [20,21], we collected in the training corpus the most frequent head nouns referring to bacteria. This resulted in the following patterns: *This (bacterium|bacteria|organism|genus|species|strain) *and *This group of organisms*, whose occurrences are considered to refer to the last bacterium.

• Generic sentence-initial definite descriptions of bacteria including a definite determiner (*The (bacterium|bacteria|organism)*) are considered to refer to the focus bacterium. The reasoning here is that because they do not use a demonstrative adjective, they instruct the reader not to consider the last bacterium; and because they are not more specific about a particular species or strain, they enforce no other particular constraint on their antecedent, so default to the current focus.

• More specific sentence-initial definite descriptions of bacteria including a definite determiner (*The (genus|species|strain)*) enforce a particular constraint on their antecedent: not only being any kind of bacterium, but more specifically being a *genus*, or a *species*, or a *strain*. They should thus select a focus bacterium which has the specified property. However, this selection of a specific antecedent was not implemented at the time of submission, and was replaced with the simple choice of the last bacterium.

• Because they are more ambiguous, non-sentence initial referring expressions (*(the|this) (organism|bacterium|bacteria)*) were not considered.

Restricting the type of antecedent to Bacteria entity mentions for the selected demonstrative and definite noun phrases is similar to the filtering of antecedents of demonstrative noun phrases implemented in [19] or to the semantic type classification of [21]. The latter mention that not using such constraints on semantic types causes a loss of 53 points in precision and 39 points in F-measure.

The co-reference resolution module takes as input the entity mentions found by the Mention identification module; it can also be tested on gold mentions.

Similarity-based co-reference resolution is run first and only uses these pre-computed mentions. It processes them in the order of the text; for each mention, it finds each anterior mention of the same type which either is identical, obeys the strain matching rule or the name matching rule, or differs by at most *e *edit operations among character insertion, deletion, substitution or swapping (*e *has been set to 1 to optimize precision).

Anaphora-based co-reference resolution is run next: It scans the text from start to end, looking for instances of referring expressions as defined above. For each referring expression, it determines an antecedent (last bacterium or focus bacterium) as explained above.

After co-referring mention pairs are thus recorded by the two strategies, transitive closure is applied to compute equivalence classes, thus forming co-reference chains including some of the input mentions and possibly the added referring expressions.

### Relationships identification

Let us recall first that the problem to solve at this stage must be managed at two levels:

**The knowledge level **with domain entities and *relations between these entities*; and

**The information level **with *instances of relations between mentions *of these entities found in the texts. For convenience we shall generally abbreviate this term as *relation instance*.

Thus we need to detect relations between entities (knowledge acquisition) based on the observation of relation instances (information extraction). Moreover, the gold standard in the training and test corpora is specified through relation instances between entity mentions (information level) which are meant to represent relations between entities (knowledge level). Evaluation is performed at the knowledge level by taking into account gold co-reference chains, as explained by the task organizers [4]; these co-reference chains are *not *provided with the test corpus.

Ideally we want to address this situation by embedding a classical information extraction framework, which learns to detect relation instances between two entity mentions based on a Maximum Entropy framework, within the knowledge acquisition framework:

**From knowledge to information: **In the training phase, we use the input mentions, the co-reference chains, and the gold relation instances to generate positive and negative examples of relation instances.

**Information extraction: **We learn to detect relation instances, making independent predictions for each of them.

**From information to knowledge: **After inference, the detected relation instances, the input mentions

and the co-reference chains should be used to make predictions at the knowledge level about entity relations.

**Description of knowledge as information: **A subset of relation instances should be selected to express each predicted relation.

However, in the last two steps, we found out that because the co-reference chains produced by our current implementation and the gold co-reference chains used to score the relations are not reliable and/or complete enough, it is difficult in the current settings to setup a clean strategy for these steps. Therefore, after multiple experiments, we decided to keep all relation instances except those involving a mention created by the anaphora resolution strategy of the co-reference resolution component: these mentions were replaced by a pre-existing co-referring mention of the same co-reference chain. Finding better strategies is left for future work.

We first define the set of Possible candidate relation instances P  as the set of relation instances between any pair of input mentions whose entity types respect the signatures of these relations. In the present domain, the relation signatures are the following:

• PartOf: Habitat*→*Habitat

• Localization: Bacteria*→*Geographical

• Localization: Bacteria*→*Habitat

The set G⊂P is the set of Gold relation instances augmented through transitive closure by the equivalence classes found by the co-reference module.  G is a very small subset of  P, even using the equivalence classes. This leads to a strong unbalance between positive and negative examples and hence to a strong bias toward negative labels, which produces models with a poor recall. To overcome this, we first restrict  P to the subset Ps⊂P of relation instances between entity mentions whose distance is smaller than a threshold *s*, where *s *is measured in sentences (more precisely, *s *is the absolute value of the difference between their sentence indexes, which is 0 when in the same sentence, 1 when in two adjoining sentences, etc.). The rationale is that positive examples are denser when entity mentions are closer to each other. Co-reference links amplify this and reduce the number of missed relation instances.

The training corpus is then constituted of all the positive examples from G  and of a set of negative examples of the same size, randomly selected from Ps\G. Since the set of negative examples is defined as the complementary of positive examples, if coreferences are not taken into account, some positive examples can potentially be chosen as negative ones in the training corpus. This would be the case for instance in Figure 3, where *C. coli *is not explicitly linked to habitats *pigs, birds*, and *surface water*: without the co-reference links, the pairs {*C. coli, pigs*}, {*C. coli, birds*}, and {*C. coli, surface water*} would be counted as negative examples. With the co-reference link between *Campylobacter coli *and *C. coli*, the gold standard Localization relations between *Campylobacter coli *and its habitats are propagated to *C. coli*, and the three above-mentioned pairs become positive examples. In our experiments, we observed that this concerns 4.1% of examples, initially considered as negative by our baseline system.

The information extraction Maximum Entropy model is trained with the following features:

• The *base *features include the words within each entity mention, as a sequence, as well as as a bag of words; the type of each entity; and the distance in sentences between them;

• The *pos *features include the same features with POS-tags instead of words;

• The *cocoa *categories, also used for entities identification;

• The *n-context *features include words and POS-tags from *n *positions on each side of each entity mention.

The development of the system requires selecting the best set of features and, if needed, context size *n*, as well as finding best distances *s *to select examples to build the train and test corpora and to tune the model regularization. Our experiments are reported in the next section.

## Results and Discussion

### Identification of bacteria and biotopes

Table 2 presents the results we achieved for mention identification on the development corpus, depending on the feature sets we selected when building the model. Mention detection was not defined as a task in itself in BioNLP-ST 2013 Bacteria Biotopes, hence no scoring was available for mention detection over the test corpus, which remains held out behind the evaluation server. All experiments were performed using the same CRF optimization parameters. These experiments are the following:

**Table 2:** Impact of selected feature sets on mention identification

	Development											
	
	Bacteria			Geographical			Habitat			Overall		
	
	R	P	F	R	P	F	R	P	F	R	P	F
Baseline (BL)	0.683	0.884	0.642	0.507	0.864	0.639	0.328	**0.892**	0.479	0.503	0.885	0.642
BL + NCBI	**0.887**	0.927	0.907	0.493	0.822	0.617	0.343	0.879	0.493	0.603	**0.907**	0.725
BL + clusters	0.811	0.913	0.859	0.493	**0.925**	0.644	0.443	0.870	0.587	0.616	0.898	0.731
BL + Cocoa	0.813	0.882	0.846	0.667	0.877	0.758	0.515	0.850	0.642	0.663	0.870	0.752
BL + OntoBiotope	0.739	0.907	0.815	0.560	0.793	0.656	0.695	0.771	0.731	0.706	0.832	0.764

**All**	**0.887**	**0.938**	**0.912**	**0.707**	0.855	**0.774**	0.705	0.829	**0.762**	**0.789**	0.884	**0.834**

All w/o clusters	0.872	0.931	0.901	0.667	0.794	0.725	0.691	0.833	0.756	0.773	0.879	0.822
All w/o Cocoa	0.872	0.937	0.903	0.573	0.843	0.683	**0.712**	0.807	0.757	0.776	0.871	0.821
All w/o NCBI	0.842	0.915	0.877	0.680	0.823	0.745	0.691	0.845	0.760	0.760	0.878	0.815
All w/o OntoBiotope	0.879	0.934	0.906	0.627	0.810	0.707	0.564	0.837	0.674	0.714	0.888	0.791

Impact of selected feature sets on mention identification. The best results are highlighted in bold

• our baseline only includes surface features (i.e., the token itself, the case of the token, the presence of punctuation in the token, the presence of digits in the token, and the length of the token);

• our baseline plus one set of features among the following four sets:

- the clusters produced with Brown's [35] algorithm;

- the NCBI taxonomy;

- the OntoBiotope ontology;

- the Cocoa annotations.

• optimal configuration: the baseline and all four features sets;

• optimal configuration minus one set of features: we removed either the Brown clusters, the NCBI taxonomy, the OntoBiotope ontology, or the Cocoa annotations.

The evaluation of the mention identification component was performed using the *conlleval.pl *script (http://www.clips.ua.ac.be/conll2000/chunking/) [41] created to evaluate results in the CoNLL-2000 Shared Task. We achieved a global F-measure ranging from 0.642 with our baseline features to 0.834 using the optimal configuration. The feature sets that increase the results are the following ones, in ascending order of the obtained gain: the NCBI taxonomy (+8.3 points), the Brown clusters (+8.9 points), the Cocoa annotations (+11 points) and the OntoBiotope ontology (+12.2 points).

We noticed that the optimal configuration generally maximizes the scores on all metrics for each category, except for precision which is better on *Geographical *(0.925*>*0.855) using the baseline and the Brown clusters only and on *Habitat *(0.870*>*0.829) using the baseline; nevertheless, in these two cases, recall is very low (respectively 21.4 and 26.2 points less) compared to that of the optimal configuration.

When analyzing the feature sets that make the system lose points with respect to the optimal configuration, we observed that not using the Cocoa annotations allowed us to slightly increase the recall of the *Habitat *category (0.712*>*0.705). However, this decreases the corresponding overall results. Removing any other feature set produced lower results than the optimal configuration.

### Co-reference resolution

#### Scoring Similarity and Anaphora strategies

In the 52 documents of the training corpus, based on gold entity mentions, our Similarity strategy computed 252 co-reference chains involving 908 mentions, whereas our Anaphora strategy computed 45 co-reference chains involving 99 mentions (see Table 3). In the 26 documents of the development corpus, Similarity found 169 co-reference chains with 589 mentions, and Anaphora found 25 co-reference chains with 52 mentions. We can thus observe that, not presuming their quality, our Similarity co-reference resolution strategy builds 5 to 7 times more co-reference chains than the Anaphora strategy.

**Table 3:** Co-reference counts on training and development corpora

		Training	Development
	documents	52	26

sim	chains	252	169
	mentions	908	589

ana	chains	45	25
	mentions	99	52

Number of co-reference chains and of entity mentions linked by these chains in the training and development corpora. sim = Similarity-based co-reference resolution, ana = anaphora-based co-reference resolution

The question we want to address here is how good is our co-reference resolution component. Since this component has been designed to help the global task of relation extraction, this should be examined from an extrinsic point of view though its impact on relation extraction. This evaluation will be performed in the next subsection.

However, the performance of relation extraction depends on a number of factors which themselves interact with the behavior of co-reference resolution. Therefore, it would be useful to obtain an evaluation of co-reference resolution from an intrinsic point of view too, so that most of its parameters can be tuned before they interact in complex ways with the relation detection component.

The most obvious way to evaluate the co-reference chains predicted by this component is to match them against those provided with the training and development corpora (no co-reference annotation was provided with the test corpus). Examination of these gold co-reference relations revealed though what we take as missing co-reference relations (some examples are provided in the discussion below). It seems therefore difficult to obtain a fair intrinsic evaluation of the co-reference resolution module. Nevertheless, for want of a better solution, we did perform this intrinsic evaluation by comparing the predicted co-reference chains against the gold co-reference annotations.

Comparing co-reference chains is a complex task. Among the reasons for this state of affairs are the fact that small changes in co-reference relations can break or merge chains and hence have a strong impact on naïve evaluation measures, the fact the task does not require to normalize the chains to a canonical reference entity which could be matched unambiguously with a gold entity, and the consideration given to "singleton" entities (non-co-referring mentions). Therefore there is no ideal method to compare co-reference chains, and a variety of scores have been designed and are usually showed together.

We have used version 7 of the scorer initially designed for the CoNLL-2011 co-reference resolution task (dated Dec 2013 on the web site but 2013-10-30 on the file. Last news and code available at http://conll.cemantix.org/2011/software.html and http://conll.github.io/reference-coreference-scorers/). It implements what is presented as consensual versions of MUC, B-CUBED and CEAF (entity based and mention based). BLANC is currently being revised and will be included in a next release.

This scorer compares predicted co-reference chains to gold co-reference chains. It implements four methods (MUC, B^3^, and CEAF using mention-based similarity or using entity-based similarity), each of which produces recall, precision and F-measure scores. For details on these co-reference evaluation measures, please see the previous links.

We evaluate the two co-reference resolution methods we have implemented: similarity of form (sim) and anaphora (ana), and their union (all). For reference, we also evaluate the application of the off-the-shelf version of the Reconcile system (rec) [18] presented in the Methods section.

Results are computed on the training and development corpora, using the gold mentions as input (Task 2). Table 4 shows that the Similarity strategy, with a recall in the range [0.835, 0.928] (training corpus) or [0.937, 0.987] (development corpus), finds nearly all the co-reference relations present in the gold annotations. However, its precision is lower on both corpora: this may correspond partly to erroneous co-reference relations and partly to missing co-reference relations in the gold annotations (we return to this point in the discussion below).

**Table 4:** Impact of co-reference strategy on co-reference resolution (all documents)

		With gold mentions						With predicted mentions		
		
		Training			Development			Development		
		
		R	P	F	R	P	F	R	P	F
sim	bcube	0.927	0.601	**0.729**	0.980	0.532	**0.690**	0.824	0.549	**0.659**
	ceafe	0.835	0.512	**0.635**	0.937	0.468	**0.624**	0.807	0.437	**0.567**
	ceafm	0.928	0.635	**0.754**	0.974	0.562	**0.713**	0.872	0.580	**0.697**
	muc	0.950	0.669	**0.785**	0.987	0.592	**0.740**	0.878	0.620	**0.727**

ana	bcube	0.006	0.059	0.011	0.012	0.100	0.021	0.009	0.075	0.016
	ceafe	0.038	0.057	0.045	0.070	0.109	0.085	0.057	0.092	0.070
	ceafm	0.029	0.101	0.045	0.046	0.180	0.073	0.041	0.157	0.065
	muc	0.000	0.000	0.000	0.000	0.000	0.000	0.000	0.000	0.000

all	bcube	0.927	0.508	0.656	0.970	0.466	0.629	0.825	0.476	0.604
	ceafe	0.823	0.448	0.580	0.910	0.433	0.587	0.791	0.396	0.527
	ceafm	0.928	0.554	0.694	0.969	0.515	0.673	0.872	0.521	0.653
	muc	0.950	0.581	0.721	0.981	0.537	0.694	0.878	0.552	0.678
			
		With self-predicted mentions								
			
rec	bcube	0.187	0.068	0.099	0.206	0.075	0.110			
	ceafe	0.230	0.040	0.068	0.379	0.056	0.098			
	ceafm	0.326	0.094	0.146	0.374	0.091	0.147			
	muc	0.319	0.109	0.163	0.308	0.089	0.139			

Impact of co-reference strategy on co-reference resolution (all documents). sim = Similarity-based co-reference resolution, ana = anaphora-based co-reference resolution, all = sim ∪ ana. bcube = *B*^3^, ceafe = CEAF-entities, ceafm = CEAF-mentions, muc = MUC. Boldface highlights F-measure of best strategy. For reference, we also show the results obtained with the off-the-shelf version of Reconcile (rec)

The Anaphora strategy obtains very low precision and recall against the gold annotations. By definition, this means that very few of the co-reference relations it finds are present in the gold annotations. The low precision is largely explained by the fact that a large part of the mentions involved in these co-reference relations (e.g., *This genus, the strain*) do not obey the constraints imposed on an entity mention in the annotation guidelines, but does not necessarily mean that they are not useful to help relation detection. It is indeed also caused by some inaccurate co-reference detection, independently of the guidelines. Finally, the low recall also concurs with the quantitative observations made in the beginning of this section: the Anaphora strategy finds a much smaller number of co-references than the Similarity strategy.

Consequently, the union of similarity-based and anaphora-based co-references (all) does not improve over the similarity-based strategy alone, and its recall is even slightly lower in many instances.

#### Reconcile system

The bottom pane of Table 4 (rec) shows the results of Reconcile co-reference resolution on the same corpora. Recall, precision and F-measure values are much lower than those obtained by our Similarity strategy, albeit higher than those of our Anaphora strategy. A possible reason why Reconcile performs better than our Anaphora strategy could be that it does detect some of the easier similar mentions (e.g., mention *C. coli *co-refers with another occurrence of *C. coli *in the same text) which are part of the gold co-reference annotations. These are also detected by our Similarity strategy, but not by our Anaphora strategy which by definition mostly collects co-referring pairs that are not in the gold annotations. Reconcile does not however include features geared towards abbreviation processing: although it does detect mentions such as *Campylobacter jejuni *and *C. jejuni *(but not all of them), it rarely finds them as co-referring.

Besides these observations, the lower results of Reconcile are largely due to the fact that it performs its own mention recognition. As a consequence, it identifies only part of the Bacteria, Habitat and Geographical mentions: its recall (measured with exact match) is 0.366 on the training corpus and 0.415 on the development corpus. This is about half the recall of our mention detection component, but co-reference recall cannot be expected to grow linearly with mention recall.

Conversely, Reconcile also identifies some pronouns and other noun phrases not in the gold standard (precision: 0.105 and 0.101), which is less of a problem to detect the co-reference relations. It is therefore somewhat more relevant to compare Reconcile's co-reference resolution results to those obtained by our co-reference component based on the mentions predicted by our entity detection component instead of the gold mentions: this is the setting in Task 3.

The right pane of Table 4 (With predicted mentions) shows these results. They are only slightly lower than those we obtain on the gold mentions. This is due to the high recall and precision of our entity detection component on Bacteria mentions (see Table 2), which make up the largest part of the co-reference relations. As a result, this does not change the positioning of Reconcile co-reference resolution (with Reconcile entity detection) with respect to our results.

We can summarize and complete these observations about the performance of a generic system such as Reconcile as follows: (*i*) its mention detection should be adapted to the present task; (*ii*) its features should be extended to cope with the abbreviation specificities of Bacteria mention co-reference; (*iii*) its features should be extended with domain-specific knowledge about the hierarchical levels of bacteria mentions presented above; for instance, among 14 occurrences of *this strain *detected by Reconcile which it included in co-reference relations, most are linked to an identical *this strain *mention, while only two are linked to a Bacteria mention (one of which is correct) and can therefore possibly lead to a useful co-reference relation for the relation detection task; (*iv*) its other anaphora-type resolution capabilities may be relevant to the present task; however, as for our own system, we cannot evaluate them because of the lack of gold annotations for this type of co-reference; (*v*) after these adaptations, it should be retrained on an annotated corpus to take into account these new features. This is unfortunately not possible for the same reason.

Having run Reconcile on the training and development corpora however shows the advantages that would be brought by using an existing high-performing framework. For instance, out of 133 occurrences of pronouns *it *and *they *in the training corpus, which we decided not to handle, Reconcile detects 40 anaphoric relations. For example, it finds the correct antecedent for the example mentioned when discussing pronouns in the Co-reference resolution section (*They *in *They are ubiquitous *refers to *Vibrios*). This points at directions for further work, which would again benefit from anaphora annotation in the corpus.

In comparison though, our co-reference component processed 59 occurrences of definite noun phrase anaphoras introduced by *this *concerning a bacterium in the training corpus, while Reconcile handled very few of these.

#### Documents with co-reference annotation

Actually, we noticed that some of the texts, mostly those with lower identification numbers, have no gold co-reference annotation at all: assuming that this might be a side-effect of different periods in the annotation process, we also provide an evaluation where only documents with gold annotations are taken into account.

Table 5 shows how this changes the evaluation figures: recall is not changed because no gold mention is removed; however, for the Similarity strategy this boosts precision by 14 to 24 points (training) or by 10 to 16 points (development) depending on the evaluation measure, and similar gains are found for the other two strategies. This applies similarly to Reconcile results, whose precision is increased by 2 to 4 points (training) or 1 point (development), and consequently does not change its positioning with respect to our co-reference results.

**Table 5:** Impact of co-reference strategy on co-reference resolution (only on documents with co-reference annotations)

		With gold mentions						With predicted mentions		
		
		Training			Development			Development		
		
		R	P	F	R	P	F	R	P	F
sim	bcube	0.927	0.756	**0.833**	0.980	0.640	**0.775**	0.824	0.667	**0.737**
	ceafe	0.835	0.757	**0.794**	0.937	0.630	**0.753**	0.807	0.605	**0.692**
	ceafm	0.928	0.799	**0.859**	0.974	0.676	**0.798**	0.872	0.705	**0.780**
	muc	0.950	0.808	**0.873**	0.987	0.691	**0.813**	0.878	0.725	**0.794**

ana	bcube	0.006	0.167	0.012	0.012	0.167	0.022	0.009	0.124	0.017
	ceafe	0.038	0.151	0.060	0.070	0.170	0.099	0.057	0.147	0.082
	ceafm	0.029	0.286	0.052	0.046	0.300	0.080	0.041	0.258	0.071
	muc	0.000	0.000	0.000	0.000	0.000	0.000	0.000	0.000	0.000

all	bcube	0.927	0.695	0.794	0.970	0.574	0.721	0.825	0.592	0.689
	ceafe	0.823	0.708	0.761	0.910	0.582	0.710	0.791	0.541	0.643
	ceafm	0.928	0.758	0.834	0.969	0.634	0.767	0.872	0.649	0.744
	muc	0.950	0.766	0.848	0.981	0.646	0.779	0.878	0.668	0.759
			
		With self-predicted mentions								
			
rec	bcube	0.187	0.091	0.122	0.206	0.086	0.121			
	ceafe	0.230	0.053	0.086	0.379	0.065	0.111			
	ceafm	0.326	0.125	0.181	0.374	0.105	0.163			
	muc	0.319	0.147	0.201	0.308	0.102	0.153			

Impact of co-reference strategy on co-reference resolution (only on documents with co-reference annotations). Because no gold annotation has been removed, recall is unchanged with respect to Table 4. sim = Similarity-based co-reference resolution, ana = anaphora-based co-reference resolution, all = sim ∪ ana. bcube = *B*^3^, ceafe = CEAF-entities, ceafm = CEAF-mentions, muc = MUC. Boldface highlights F-measure of best strategy. For reference, we also show the results obtained with the off-the-shelf version of Reconcile (rec)

The incompleteness of gold co-reference annotations from the point of view of an intrinsic co-reference evaluation makes it difficult for us to assess our objective of a precision-oriented co-reference resolution component. This intrinsic evaluation characterizes it on the contrary as a high-recall component with moderate precision, but the co-reference annotations provided with the training and development corpora were not meant initially as a gold standard to evaluate co-reference resolution. The present exercise seems to reach its limits in this respect.

Finally, we also tested the impact of two parameters of the Similarity strategy, which we summarize shortly. Not blocking the single-*bacterium *mentions incurs a loss of 7 to 10 F-measure points on both training and development corpora: considering these mentions as systematically co-referent would be like considering that two occurrences of pronoun *it *are always co-referent. We also set the maximum hierarchical distance to various numbers between 0 (same level) and 10 (no constraint). More stringent constraints decreased F-measure by up to one point without gaining precision.

The best results of these parameters on the development corpus were used to determine the configuration to retain to produce the co-reference chains passed to relation detection: this led to discard the hierarchical distance constraint and to block the single-*bacterium *mentions.

### Relation detection

We evaluated relation detection using the official scoring program on the development corpus (locally) and on the test corpus (by submitting runs to the on-line evaluation server). Recall, precision and F-measure were computed for each run.

Relation detection is the overall task addressed in the present work, and as such it depends on the input it is provided (mentions and co-reference chains) as well as on its own parameters (maximum distance *s*, feature sets, including context size). The questions we examine here are the determination of its optimal set of parameters, and an extrinsic evaluation of co-reference resolution.

In all the reported experiments, only one parameter was optimized at a time and all other parameters were set to the optimal value for clarity. The optimal set of all parameters was found using an almost full grid-search on the development corpus.

**Figure 5:**
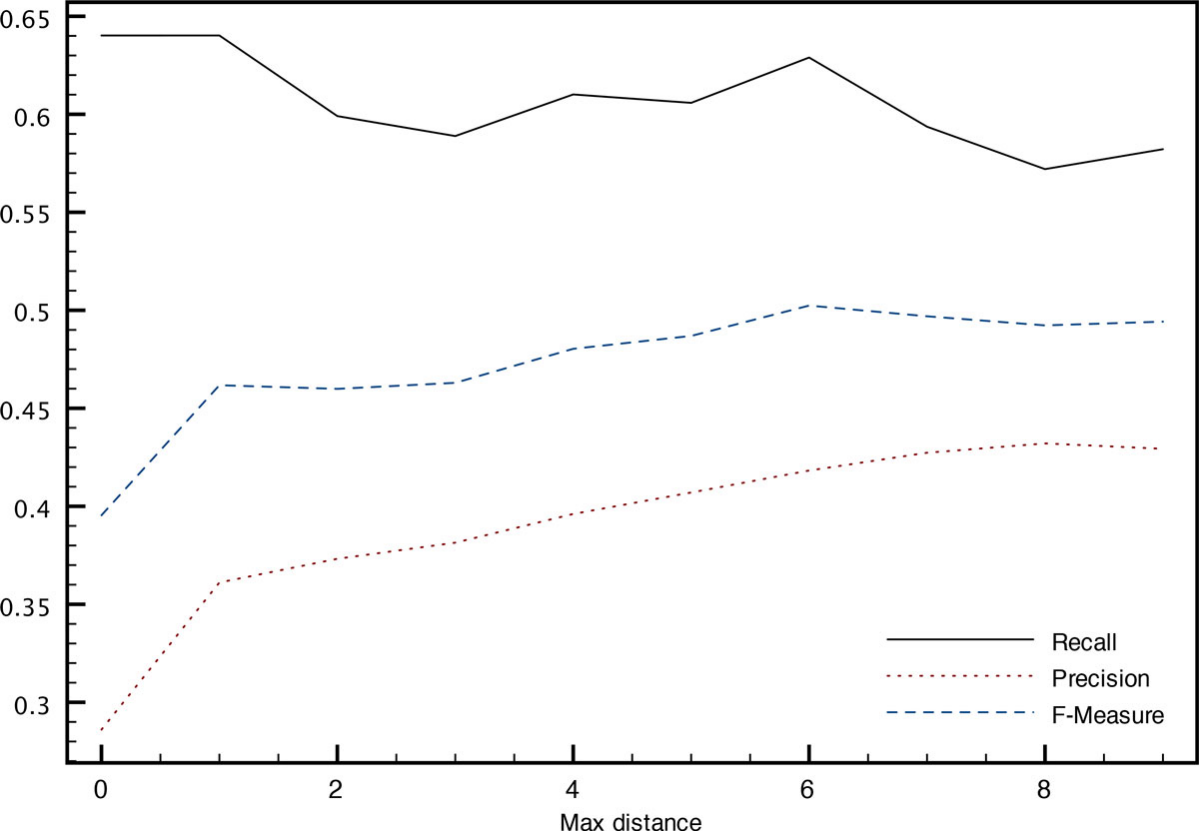
**Impact on relation detection of the maximum distance (in sentences) between entity mentions when training (training corpus)**. Variation of performance with the maximum distance *s *between entity mentions at training time.

**Figure 6:**
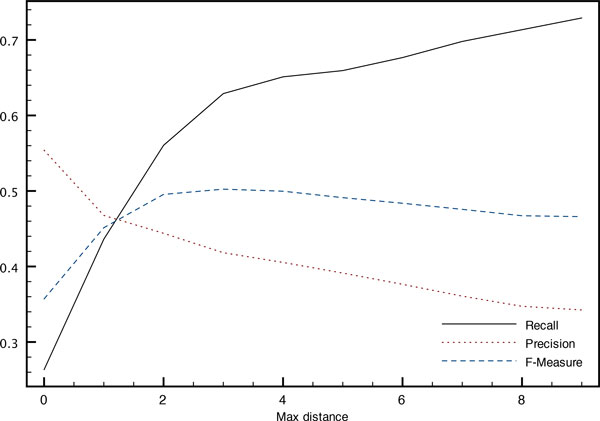
**Impact on relation detection of the maximum distance (in sentences) between entity mentions when decoding (development corpus)**. Variation of performance with the maximum distance *s *between entity mentions at decoding time.

The influence of the maximum distance *s *allowed between the two entity mentions forming a relation both while building the training corpus and at decoding time is illustrated on Figures 5 and 6. An increased distance allows the system to build a larger corpus with more variety in the examples, which leads to an increased precision of the system without too much impact on its recall.

On the other hand, at decoding time, plots are reversed. Relation instances between more distant entity mentions are sparser, leading to a decreased precision. The best F-measure is achieved at a maximum distance of *s *= 3 where 11.4% of the relations cannot be predicted even with the co-references. Increasing the distance reduces the number of missed relation instances, thereby increasing recall but at the cost of a decreased precision.

**Figure 7:**
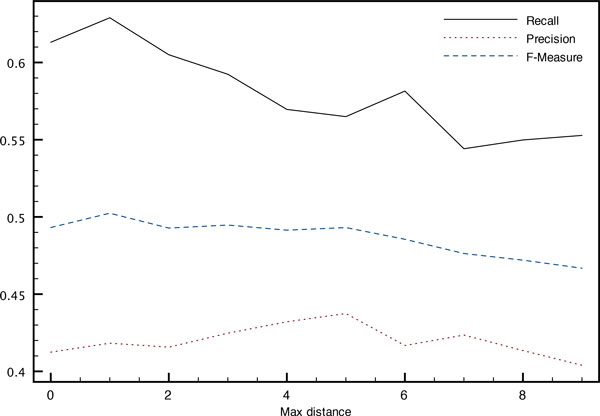
**Impact on relation detection of the size of the context surrounding each mention**. Variation of performance with the size *n *of the left and right contexts used to collect features for each entity mention on the development corpus.

The impact of the size *n *of the context of mentions in which features are collected is shown on Figure 7. Our experiments did not evidence an improvement of context sizes larger than one token.

Table 6 displays the impact of our co-reference resolution strategies on relation detection (these experiments use the full set of features, see below the discussion of Table 7). It shows that the Similarity (sim) and All strategies clearly outperform the no-co-reference baseline (none). It also confirms that the Similarity strategy obtains the best evaluation results, which is consistent with the intrinsic evaluation presented in the previous subsection. This strategy gains 7 points of F-measure over having no co-reference resolution on both development and test sets on gold mentions; and 5.5 points (development) or 3.5 points (test) on predicted mentions. This is congruent to [42], who report a 13-point loss of F-measure from 0.45 to 0.325. The system incurs a loss of 10 points in the best result when shifting from gold mentions (Task 2, 0.497) to predicted mentions (Task 3, 0.394). Recall is higher than precision with gold mentions, but recall and precision are balanced when computed over predicted mentions.

**Table 6:** Impact of co-reference resolution on relation detection

	Development			Test		
	**R**	**P**	**F**	**R**	**P**	**F**

T2: none	0.582	0.338	0.428	0.608	0.324	0.423
T2: sim	0.583	0.433	**0.497**	0.598	0.422	*0.495*
T2: ana	0.559	0.307	0.397	0.602	0.311	0.410
T2: all	0.595	0.411	0.486	0.587	0.389	0.468

T3: none	0.368	0.312	0.338	0.360	0.281	0.316
T3: sim	0.388	0.399	**0.394**	0.347	0.356	*0.351*
T3: ana	0.317	0.273	0.293	0.334	0.279	0.304
T3: all	0.391	0.367	0.379	0.333	0.357	0.345

Impact of co-reference resolution on relation detection. These experiments use the optimal (i.e., full '+context') set of features tested in Table 7, hence the best results here are optimal with respect to feature sets too. none = no co-reference resolution; sim = string similarity; ana = anaphora; all = sim+ana. Boldface highlights the optimal value on the development corpus, italics the associated value on the test corpus

**Table 7:** Impact of accumulated feature sets on relation detection

	Development			Test		
	**R**	**P**	**F**	**R**	**P**	**F**

T2: baseline	0.567	0.400	0.469	0.621	0.398	0.485
+ POS tags	0.578	0.406	0.477	0.634	0.424	0.508
+ Cocoa	0.617	0.409	0.492	0.645	0.420	0.508
+ context	0.583	0.433	**0.497**	0.598	0.422	*0.495*

T3: baseline	0.359	0.375	0.367	0.324	0.360	0.341
+ POS tags	0.374	0.386	0.380	0.322	0.385	0.351
+ Cocoa	0.396	0.386	0.391	0.332	0.376	0.352
+ context	0.388	0.399	**0.394**	0.347	0.356	*0.351*

Impact of accumulated feature sets on relation detection. These experiments use the optimal co- reference resolution strategy evidenced in Table 6, hence the best results here are optimal with respect to co-reference resolution too. Boldface highlights the optimal value on the development corpus, italics the associated value on the test corpus. By construction, highlighted values are the same as in Table 6

These observations made on the development corpus are also valid on the test corpus, a good property of our protocol since it denotes that controlled experiments on the development corpus were predictive of system behavior on the test corpus. Hence the setting selected because it was optimal on the development corpus (Similarity strategy, bold F-measures) proved to obtain the best F-measure on the test corpus (italics F-measures).

The Anaphora strategy of our co-reference component performed poorly in this extrinsic evaluation. This confirms its low recall and precision in the intrinsic evaluation presented in Tables 4 and 5 and the much smaller number of co-reference relations that it found compared to the Similarity strategy. While precision of relation extraction is improved by co-reference resolution, recall is not. We hypothesize that this comes from the changes that co-reference resolution induces in the generation of training examples.

The system generates positive examples from the relations found in the training corpus. As explained in Section Relationships identification, co-reference increases the number of positive examples: our best system increases it by a factor of 4.5 from 415 (no co-reference) to 1852 (Similarity). These new positive examples were previously considered as negative, and could be selected when building the set of negative examples. This happened to 10% (39) of the negative examples of the no co-reference setting.

The fact that recall does not increase implies that the new positive examples do not provide knowledge about previously unknown relation patterns. In contrast, the fact that precision increases can be related both to the increase in training set size and to the reduction in the noise caused by incorrect negative samples.

Table 7 reports experiments on the successive addition of the four feature sets defined in the Methods section, using the optimal co-reference strategy, i.e., Similarity (see above and Table 6). While this monotonically improves the results on the development set (maximum F-measure in bold), this is not the case on the test set, and we end up selecting the full feature set, which happens not to be optimal on the test set (associated F-measure in italics), losing 1.3 F-measure points on the gold mentions and 0.1 point on the predicted mentions when adding context features.

### Discussion

The tables show that our best relation detection results are higher that the top performing system of BioNLP-ST 2013 [24] (TEES 2.1, F = 0.42 on Task 2, F = 0.14 on Task 3): without co-reference resolution, it is on par with it on gold mentions, and twice as high on predicted mentions; and it outperforms it by a large margin with co-reference resolution (7 points on gold mentions and 24 points on predicted mentions). However, we believe there is still room for much improvement, because of the current following limitations.

We were not able to take much advantage of the context of entity mentions, i.e., information outside the tokens that form the entity mention. This is counter-intuitive and seems to be linked to the difficulty of the system to learn enough useful clues to compensate for the noise added by a more extended context.

We observed that the gold co-reference chains provided by the task organizers are not complete with respect to the needs of relation instance detection as defined above. In contrast to some clinical data sets [8,9], they never include pronouns (*it, they, its*) or demonstrative adjectives (*this*), which are nonetheless instrumental to linking locations to bacteria in a number of instances: see for instance *This species *in Figure 2, which links the bacterium of the previous sentence to its localizations in the current sentence.

Generic noun phrase anaphoras such as *Isolated strains *in *The genus [Spirochaeta] represents a group of . . . Isolated strains have been obtained from a variety of [freshwaters]* . . . are not annotated either, although they provide the link to a Localization relation *Spirochaeta−*(loc)*→freshwaters *which is annotated in the gold standard.

Co-reference sometimes also percolates through sortal relations in constructions such as *The genus [Burkholderia] consists of some 35 [bacterial species], most of which are [soil] saprophytes*, where *bacterial species *is not strictly speaking co-referring with *Burkholderia *(it is instead an element or subclass of genus *Burkholderia*), but still supports the inference that *Burkholderia *is found in habitat *soil*. This points at the need for more elaborate inference paths to identify localization relations.

A recurring example is that of diseases [42] which we also identified in the training corpus: bacteria often cause diseases in hosts (e.g. in humans), hence the path *bacteria−*(causes)*→disease *+ *disease−*(affects)*→host*, which relies on the detection of two relation legs, offers strong support to the establishment of a localization relation *bacteria−*(loc)*→host*.

Future work includes short-term easy steps and longer-term steps. We used word clusters as additional features for mention detection, it will be straightforward to test them in relation detection: they might bring useful generalizations for context features. Manual collection of features [42] is a complementary option here.

Longer-term investigations are needed to discuss and design a more complete gold annotation of co-reference chains. We consider this is a precondition to design effective strategies for going up from information extraction to the knowledge level and to represent relations by suitable relation instances. This will also make it possible to train machine-learning co-reference methods, including building upon existing frameworks such as Reconcile [17,18].

## Conclusions

The methods tested here and the reported experiments confirm that accurate co-reference resolution is key to accurate relation detection at the knowledge level in the present type of corpus. Co-reference chains first play a role in the generation of positive and negative examples of relation instances, hence on the training phase of supervised relation detection. Additional experiments, not detailed here for reasons of space, show that if co-reference chains are not used when training, the gain of using them only at the inference stage is only 0.6 F-measure point, which is 10 times less than the 5.5 to 7 F-measure points gained when using co-reference chains at both stages. Second, they play a role in the management of the detected relation instances. Third, they are used by the evaluation program to assess the accuracy of the proposed relation instances as valid representatives of relations between entities.

We designed and included in our relation detection system a co-reference resolution component which is close in spirit to that of [42]. This component contributes from 3.5 (test) to 5.5 F-measure points (development) when run on mentions predicted by our mention detection component, and 7 F-measure points (test and development) with gold mentions. Its predicted co-reference chains are used in the first and second steps, but the gold co-reference chains used in the evaluation step are those prepared by the task organizers. This makes it important for this step that predicted co-reference chains be close to gold co-reference chains, but also that gold co-reference chains be accurate and complete. However, we have discussed some limitations in the present gold co-reference chains which we believe need to be overcome for more progress to be made on relation extraction on this type of documents.

The methods presented here and tested on the official BioNLP-ST 2013 test corpora achieve a 0.495 F-measure taking as input the gold entity mentions, and a 0.351 F-measure when taking as input entity mentions predicted by our CRF system. This outperforms the best results obtained on this corpus in the challenge (F = 0.42 or 0.14) and thus provides new baselines for the task. We believe a better treatment of co-reference, obtained through a more complete gold annotation of anaphora and other co-referring expressions, should make it possible to identify more relevant features for the type of co-reference-mediated relation detection encountered in the present type of corpus.

## Competing interests

The authors declare that they have no competing interests.

## Authors' contributions

All authors designed the experiments, wrote, read and approved the paper. CG prepared the mention detection module and a first version of the relation detection module, PZ prepared the co-reference module, and TL prepared the final relation detection module.

## Declarations

The publication costs of this article were funded by project Accordys (*Agrégation de Contenus et de COnnaissances pour Raisonner à partir de cas de DYSmorphologie fœtale*, Content and Knowledge Aggregation for Case-based Reasoning in the field of Fetal Dysmorphology (ANR 2012-2015)) funded by ANR under grant number ANR-12-CORD-0007-03.

This article has been published as part of *BMC Bioinformatics *Volume 16 Supplement 10, 2015: BioNLP Shared Task 2013: Part 1. The full contents of the supplement are available online at http://www.biomedcentral.com/bmcbioinformatics/supplements/16/S10.
